# Diagnostic significance of calf circumference in sarcopenia of healthy korean adult males

**DOI:** 10.3389/fphys.2022.973265

**Published:** 2022-09-16

**Authors:** Gwon-Min Kim, Seunghwan Song, Jong-Hwan Park, Young Jin Tak, Il Jae Wang, Up Huh, Jeong Su Cho

**Affiliations:** ^1^ Department of Medical Research Institute, Pusan National University, Busan, South Korea; ^2^ Department of Thoracic and Cardiovascular Surgery, Pusan National University School of Medicine, Biomedical Research Institute, Pusan National University Hospital, Busan, South Korea; ^3^ Health Convergence Medicine Laboratory, Biomedical Research Institute, Pusan National University Hospital, Busan, South Korea; ^4^ Department of Family Medicine, Pusan National University School of Medicine, Busan, South Korea; ^5^ Department of Emergency Medicine, Pusan National University School of Medicine, Biomedical Research Institute, Pusan National University Hospital, Busan, South Korea

**Keywords:** sarcopenia, computed tomography, calf circumference, skeletal muscle mass index, obesity

## Abstract

This study aimed to determine the relationship between sarcopenia and physical function, and the best method of diagnosing sarcopenia in healthy adults. Early prevention of sarcopenia is important; however, no studies have been conducted in healthy and relatively young adults. In addition, it remains unclear whether calf circumference is associated with sarcopenia-defined variables. A total of 85 healthy male participants were enrolled, and the mean standard deviation age of the participants was 59.76 (8.12) years. Abdominal computed tomography (CT) was performed to measure muscle areas. All subjects were divided into sarcopenia and non-sarcopenia groups based on skeletal muscle mass index using computed tomography. Sarcopenia showed a tendency to be related to lower grip strength, five times sit-to-stand and timed up and go tests for physical function. This result shows that overweight and obesity in the sarcopenia group had fully adjusted odds ratios of 0.026 (95% CI: 0.002–0.317) and 0.008 (95% CI: 0.001–0.096), respectively. Calf circumference was higher specificity (71.43 and 64.86) better than bioelectrical impedance analysis-based skeletal mass index and had a similar sensitivity (72.09 and 82.35). In conclusion, calf circumference suggests the need to consider its use as a tool for assessing muscle mass in the diagnosis of sarcopenia.

## 1 Introduction

A new definition of sarcopenia was proposed by the European Working Group in Older People (EWGSOP) (Cruz-Jentoft et al., 2010). Since then, sarcopenia research has skyrocketed worldwide, including Asia (Rosenberg, 1997). However, diagnosing sarcopenia in Asian populations requires special consideration because of cultural and/or lifestyle-related differences (Baumgartner et al., 1998). The Asian Working Group for Sarcopenia (AWGS) has invigorated sarcopenia research in Asia, and their cut-off is widely used in Asia. These groups use different cut-offs to define sarcopenia in aging, highlighting the fact that different cut-offs are necessary for different ethnic groups (Chen et al., 2014).

In recent research on sarcopenia, most definitions state that a person with lower muscle strength and lower muscle quality or mass should be diagnosed with sarcopenia. Sarcopenia is directly responsible for reduced strength, which elevates the risk of negative outcomes, such as decreased physical function (Janssen et al., 2002; Newman et al., 2003). Obesity may aggravate sarcopenia in the elderly and maximize its effects on mortality, morbidity, and physical dysfunction (Zamboni et al., 2008; Kalinkovich and Livshits, 2017). One study confirmed that serum high-density lipoprotein cholesterol (HDL-C) and triglyceride (TG) levels had negative and positive relationships with sarcopenia, respectively (Du et al., 2018). The serum testosterone index is significantly associated with arm and leg strength and fat-free mass in generally healthy men (Baumgartner et al., 1999).

Muscle mass can be measured using dual-energy X-ray absorptiometry (DXA), magnetic resonance imaging (MRI), computed tomography (CT), and bioimpedance. CT is used for the evaluation of skeletal muscle mass and may be a useful method for defining sarcopenia (Beaudart et al.). The transverse skeletal muscle area at the third lumbar vertebra (L3) level has been shown to correlate strongly with body muscle distribution (Shen et al., 2004; Fearon et al., 2011). A previous study suggested that calf circumference may also be used to assess muscle mass (Evans et al., 1995; Baumgartner et al., 1998). A recent study found that calf circumference significantly relates to skeletal muscle mass (González-Correa et al., 2020). However, calf circumference has not still assessed as a screening tool for sarcopenia in the elderly population.

Thus, although sarcopenia has been reported in many older adults, it remains unclear whether there is an association between sarcopenia and healthy or young adults. In addition, it remains unclear whether calf circumference is associated with sarcopenia-defined variables. Early prevention of sarcopenia is important; however, no studies have been conducted in healthy and relatively young adults. This study aimed to determine the relationship between sarcopenia and physical function, and the best method of diagnosing sarcopenia in healthy adults.

## 2 Methods

### 2.1 Study design and participants

**FIGURE 1:**
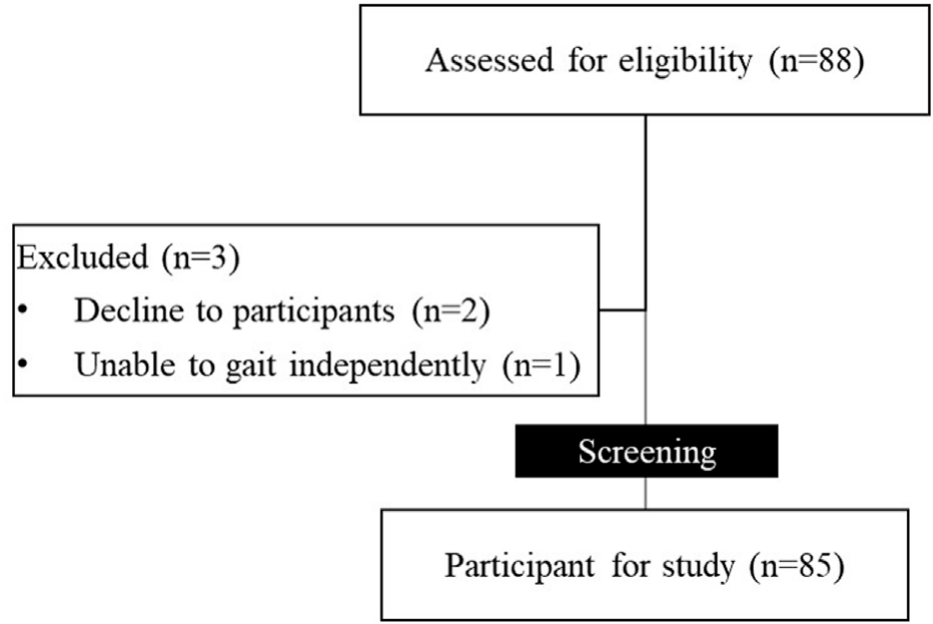
Study flow diagram of participant enrolment.

Participants were enrolled in this study between September 2020 and August 2021 at Pusan National University Hospital, based on the diagram shown in Figure 1. Finally, 85 participants, including only healthy without the disease male adults, were enrolled. Decline to participants and unable to gait independently were excluded. Abdominal CT was performed to measure the muscle area. This study was approved by the Research Ethics Committee of the Pusan National University Hospital (2008–005-093). All participants were provided with a detailed description of the experiment, and written informed consent was obtained prior to participation, in accordance with the ethical standards of the Declaration of Helsinki. All methods were performed in accordance with the approved study plan, as well as with relevant guidelines and regulations.

### 2.2 CT-based skeletal muscle index and sarcopenia definition

**FIGURE 2:**
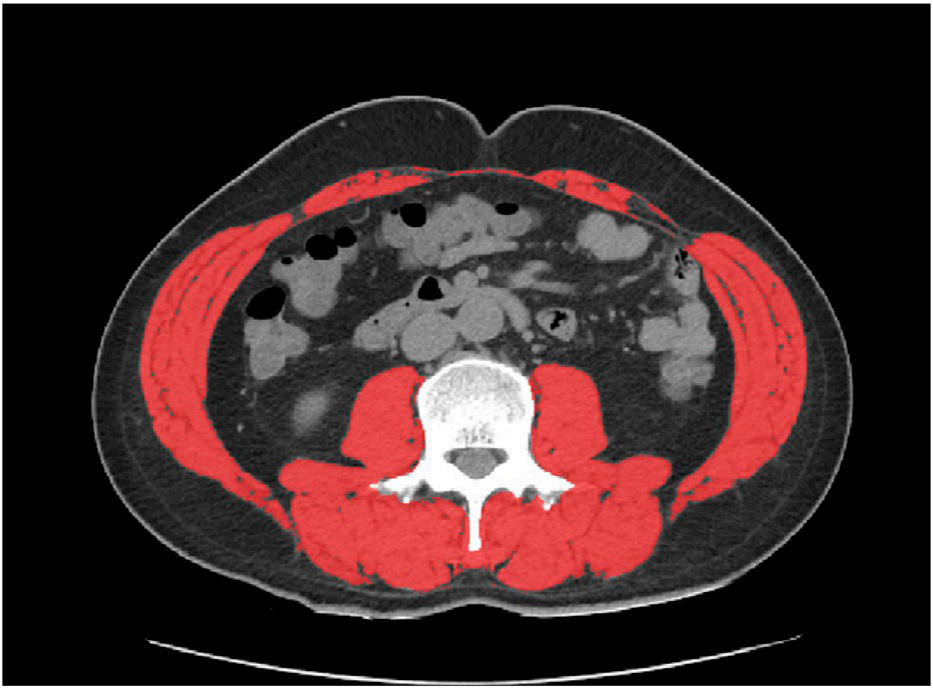
CT images of L3 skeletal muscle area measurement (red area represents skeletal muscle area).

Abdominal CT image analyses were performed on a dedicated post-processing website (Core-slicer, https://coreslicer.com/) using cross-sectional images at the level of the mid-third lumbar vertebra (Figure 2). The total muscle area and visceral adipose tissue (VAT) were determined in terms of area (cm^2^) and mean attenuation (Hounsfield units, HU), respectively. We used additional density thresholds (−45 to +130 HU) to detect muscle and exclude intramuscular and adipose tissue areas for muscle area segmentation. In this study, the definition of sarcopenia was set at <53.0 cm^2^/m^2^ (Montano-Loza et al., 2016), and the skeletal muscle area was converted to the skeletal muscle index (SMI, cm^2^/m^2^).

### 2.3 Body composition and calf circumference

Body mass index and appendicular skeletal muscle (ASM) mass index were measured using bioelectrical impedance analysis (BIA). Body composition was determined using an InBody 770 device (Inbody, Seoul, Korea). The BIA-based SMI was calculated as ASM/height^2^ (Baumgartner et al., 1998). Calf circumference was measured using tape around the largest part of the calf to the nearest millimeter, with participants in the seated position and the calf placed at a right angle to the thigh (Tang et al., 2020).

### 2.4 Physical function test

Grip strength was measured using a handheld dynamometer (TKK 5401; Takei Scientific Instruments, Tokyo, Japan) placed in the dominant hand of the participants. During the assessment, participants were asked to stand upright with their elbow fully extended and their feet shoulder-width apart. The test was repeated and the mean value was recorded. In the five times sit-to-stand test (FTSS), participants were asked to sit down and stand up five times as fast as possible with their arms crossed over their chest; the results were measured in seconds (Walrand et al., 2011). For the timed up-and-go test (TUG), a 3-m walkway was marked on a flat surface using a cone on the floor; the starting chair had no armrests but did have a back support. Participants were then asked to stand up from the chair, walk forward for a 3-m distance as rapidly as possible, and return to sit. FTSS and TUG test using an armless chair with the seat 43 cm from the floor. Gait speed was measured for a normal speed gait, with the units measured in seconds. The gait distance was measured using a 7-m walkway after excluding a 1.5-m acceleration and deceleration zone at either end, leaving a total measurement distance of 4 m. Physical activity was assessed using the Korean version of international physical activity questionnaire-Short version (IPAQ-S) (Oh et al., 2007). The IPAQ-S asks about physical activity and sedentary time. The structured items in the IPAQ-S provide separate scores for sedentary, walking, moderate-intensity, and vigorous-intensity activities. Moderate-to-vigorous physical activity and sedentary time were used in analyses.

### 2.5 Blood pressure and blood test

Arterial blood pressure was measured in the right arm with the participant in a seated position using a blood pressure monitor (HEM-7121; Omron Corporation, Kyoto, Japan). Blood analysis was performed on plasma samples (5 ml) obtained from the participants. A blood sample was taken from the antecubital vein of the participants after an 8-h fast. The samples were collected in a seated position according to a protocol and were centrifuged within 40 min of collection. The samples were analyzed using an autoanalyzer (Hitachi 902; Roche, Manheim, Germany). Triglyceride (TG), total cholesterol (TC), high-density lipoprotein cholesterol (HDL-C), low-density lipoprotein cholesterol (LDL-C), testosterone, and creatinine levels were analyzed as standard lipid profiles.

### 2.6 Statistical analysis

Data analysis was performed using SPSS for Windows (version 21; SPSS Inc., Chicago, IL, United States). The characteristics of the study population are presented as mean ± standard deviation (SD) for continuous variables and as frequencies and proportions for categorical variables. Chi-squared statistics were used for hypothesis testing as appropriate. Pearson’s correlation coefficient was used to evaluate the degree of correlation between variables. Student’s t-test was performed to compare the measures and sarcopenia differences between the two groups. Logistic regression analysis was used for BMI that were significantly different in the non-sarcopenia and sarcopenia comparison. Model 1 was not adjusted and model 2 was adjusted for age, grip strength, FTSS, TUG, gait speed, MVPA, and sedentary time. Model 3 was adjusted for age and TG, TC, LDL-C, HDL-C, testosterone, and creatinine levels. Model 4 was adjusted for potential confounders, including variables in models 2 and 3. A *p*-value less than 0.05 was considered significant. The three values to define sarcopenia for the two groups were compared using receiver operating characteristic (ROC) analysis using the area under the ROC curve (AUC) and confidence interval. Analysis was conducted using MedCalc for Windows ver. 9.1.0.1 (MedCalc^®^ Corp, Mariakerke Ostend, Belgium).

## 3 Results

Table 1 shows the participants’ baseline characteristics and biochemical variables. The mean (SD) age of the participants was 59.76 (8.12) years, and 100% were male. Regarding physical results, the mean grip strength, FTSS, TUG, gait speed, MVPA, and sedentary activity were 35.57, 7.18, 5.57, 1.21, 198.69, and 414.29, respectively (SDs: 6.12, 1.83, 1.05, 0.15, 220.40, and 192.06, respectively), and regarding biochemical variables, the mean testosterone, creatinine, TG, TC, LDL, and HDL levels were 4.23, 1.01, 96.49, 179.99, 125.13, and 54.30, respectively (SDs: 1.93, 0.1, 56.86, 31.98, 29.57, 12.85, respectively). There were no significant differences in any of the variables between sarcopenia groups. In the non-sarcopenia and sarcopenia group, the mean BMI was 26.10 (SD: 1.98) kg/m^2^ and 23.20 (2.22) kg/m^2^, respectively; the mean calf circumference was 39.61 (2.20) cm and 37.30 (2.97) cm, respectively; significant differences between the sarcopenia groups were identified for all variables.

**TABLE 1:** Baseline characteristics and biochemical variables in participants.

	Total (85)	Non-sarcopenia (42)	Sarcopenia (43)	*p*
Age (years)	59.76 ± 8.12	59.10 ± 7.07	60.42 ± 9.06	0.209
BMI (kg/m^2^)	24.63 ± 2.55	26.10 ± 1.98	23.20 ± 2.22	<0.001
Height (cm)	169.84 ± 6.62	169.19 ± 5.87	170.46 ± 7.28	0.383
Weight (kg)	71.25 ± 9.61	75.03 ± 7.86	67.64 ± 9.82	<0.001
BIA-based SMI (kg/m^2^)	8.79 ± 0.70	9.08 ± 0.69	8.52 ± 0.60	<0.001
Calf circumference (cm)	38.44 ± 2.85	39.61 ± 2.20	37.30 ± 2.97	0.002
Grip strength (kg)	35.57 ± 6.12	35.34 ± 7.48	33.31 ± 8.03	0.202
FTSS (sec)	7.29 ± 1.83	7.18 ± 1.96	7.39 ± 1.70	0.929
TUG (sec)	5.61 ± 1.05	5.57 ± 1.07	5.65 ± 1.04	0.806
Gait speed (m/s)	1.20 ± 0.15	1.21 ± 0.16	1.20 ± 0.14	0.571
MVPA (min)	165.24 ± 220.40	198.69 ± 217.42	132.56 ± 220.90	0.564
Sedentary time (min)	403.06 ± 192.06	414.29 ± 190.11	392.09 ± 195.56	0.973
Biochemical variables				
Testosterone (ng/ml)	4.23 ± 1.93	4.22 ± 1.94	4.25 ± 1.94	0.274
Creatinine (mg/dl)	1.01 ± 0.15	1.02 ± 0.15	1.01 ± 0.15	0.781
TG (mg/dl)	96.49 ± 56.86	102.03 ± 54.97	91.08 ± 58.79	0.898
TC (mg/dl)	179.99 ± 31.98	181.43 ± 33.34	178.58 ± 30.93	0.478
LDL-C (mg/dl)	125.13 ± 29.57	126.99 ± 31.05	123.31 ± 28.29	0.650
HDL-C (mg/dl)	54.30 ± 12.85	52.20 ± 12.20	56.34 ± 13.27	0.054

BMI, body mass index; BIA, bioelectrical impedance analysis; SMI, skeletal muscle mass index; FTSS, five times sit-to-stand; TUG, timed up-and- go; MVPA, moderate-to-vigorous physical activity; TG, triglyceride; TC, total cholesterol; LDL-C, low-density lipoprotein cholesterol; HDL-C, high-density lipoprotein cholesterol.

Comparisons of the odds ratios for overweight and obesity in the sarcopenia group are shown in Figure 2. Participants were categorized into three groups based on their BMI: less than overweight, overweight, and obese. Model 1 showed that overweight and obesity in the sarcopenia group had odds ratios of 0.048 (95% CI: 0.005–0.413) and 0.016 (95% CI: 0.002–0.138), respectively, relative to those who were not overweight. In addition, Model 4 in the fully adjusted group had an odds ratio of 0.026 (95% CI: 0.002–0.317) for overweight and 0.008 (95% CI: 0.001–0.096) for obesity.

The relationship between CT-based SMI, calf circumference, BIA-based SMI, grip strength, and gait speed was found (Figure 4). For both muscle mass index and grip strength, gait speed was almost mutually uncorrelated, whereas CT-based SMI and calf circumference (approximately 0.48, *p* < 0.01) and CT-based SMI and BIA-based SMI (approximately 0.38, *p* < 0.01) correlated.

We observed that an optimal relationship between sensitivity and specificity was achieved at a calf circumference of 39.00 cm (sensitivity, 72.09%; specificity, 71.43%) and BIA-based SMI of 8.73 kg/m^2^ (sensitivity, 82.35%; specificity, 64.86%), which had an AUC of 0.768 (95% CI: 0.664–0.853) and 0.771 (95% CI: 0.656–0.862), respectively (Figure 5).

## 4 Discussion

We found a strong correlation between calf circumference and CT-based SMI in healthy adults with sarcopenia. In addition, calf circumference was more relevant than the BIA-based SMI in participants with sarcopenia. Calf circumference suggests the need to consider its utilization as a tool for assessing muscle mass in diagnosing sarcopenia.

Sarcopenia showed a tendency to be related to lower grip strength, FTSS, and TUG scores. However, physical function did not meet the definition of sarcopenia, and there was no significant association (Table 1). Increased fat mass might be more predictive of lower physical function and functional limitation than decreased muscle mass (Zamboni et al., 1999). Our study participants was relatively young male, thought that there would have been no difference in physical function even if the amount of muscle mass were different. Also, because it is sarcopenia, but it is not underweight.

**FIGURE 3:**
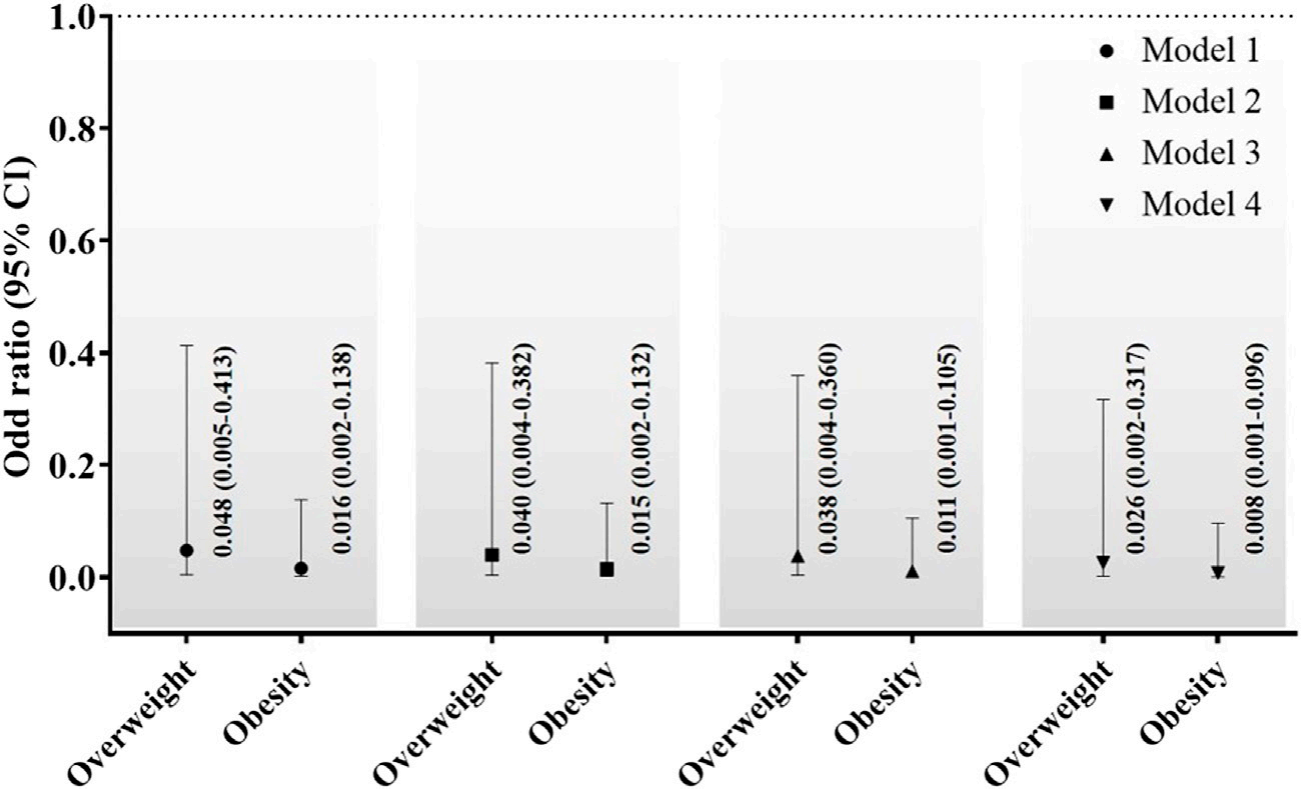
Comparisons of the odds ratios for overweight and obesity of sarcopenia group. CI; confidence interval. Model 1, no adjustments; Model 2, adjusted for age, grip strength, FTSS, TUG, gait speed, moderate-to-vigorous physical activity, and sedentary time; Model 3: adjusted for age, TG, TC, LDL-C, HDL-C, testosterone, and creatinine; and Model 4: adjusted for potential confounders including variables in Model 2 and Model 3.

Obesity is associated with sarcopenia and shares pathological mechanisms (Walrand et al., 2011). Body composition changes with aging include a decrease in skeletal muscle mass and an increase in visceral fat (Tyrovolas et al., 2016). Fat mass causes inflammation, which may increase the development of sarcopenia (Gregor and Hotamisligil, 2011). However, our research rejects the mechanisms suggested by prior studies. The results showed that overweight and obesity in the sarcopenia group had a fully adjusted odds ratio of 0.026 (95% CI: 0.002–0.317) for overweight and 0.008 (95% CI: 0.001–0.096) for obesity (Figure 3). The present findings support those of previous cross-sectional studies that identified a low prevalence of obesity and sarcopenia (Stenholm et al., 2008).

Previous studies have suggested that higher testosterone levels in older men may significantly increase leg strength as well as muscle mass (Wang et al., 2000). Another study found that total cholesterol, HDL-C, and fasting glucose were more strongly associated than LDL-C with sarcopenia (Du et al., 2017; Du and No, 2017). However, our results showed no significant differences between the non-sarcopenia and sarcopenia groups in all biochemical variables (Table 1). These results were found to be related more to fat than to muscle. A strong association between adiposity and obesity on serum lipid and lipoprotein levels has been established (Sattar et al., 1998).

**FIGURE 4:**
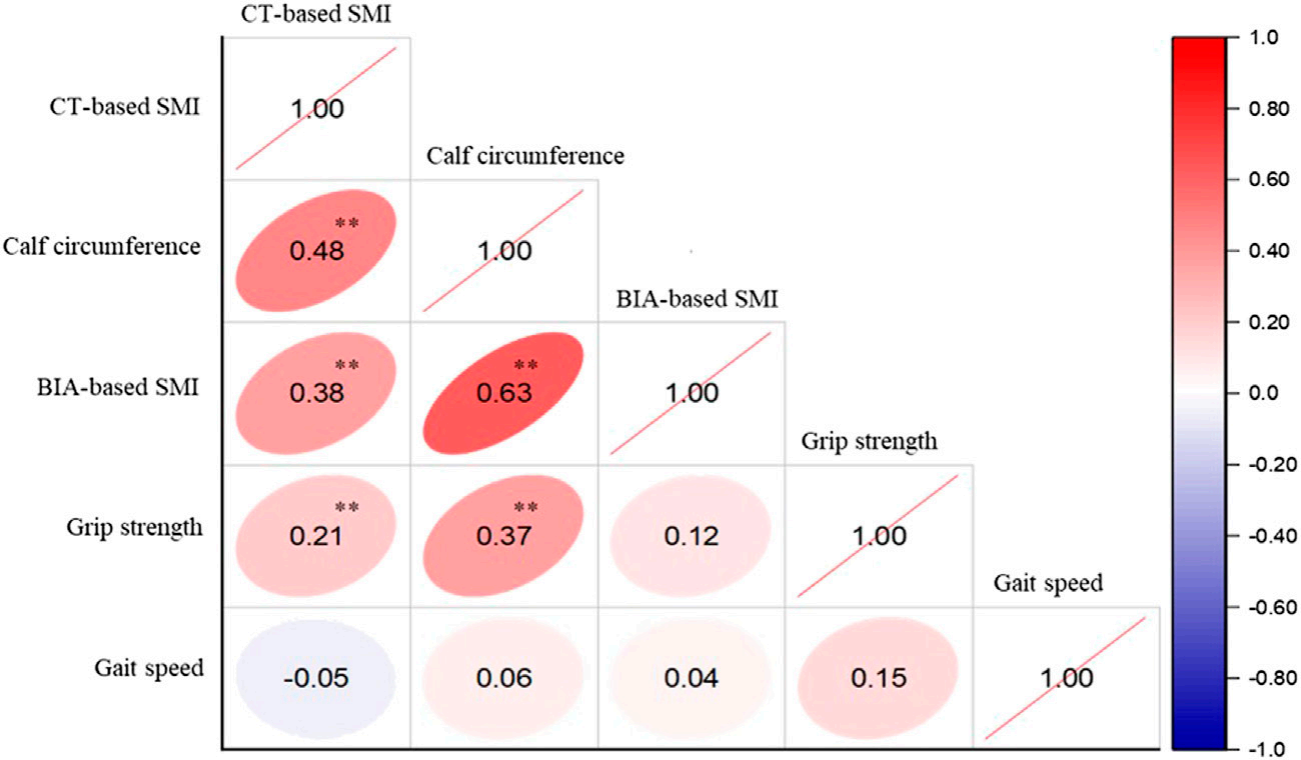
Correlation between variables related to sarcopenia definition and muscle mass index measured using CT. CT, computed tomography; SMI, skeletal muscle mass index; BIA, bioelectrical impedance analysis. **p < 0.01.

Many studies have used complicated procedures to define sarcopenia. There are various measurement and assessment methods for muscle mass, strength, and performance. CT, MRI, and DXA to assess skeletal muscle mass, might be the gold standard for defining sarcopenia (Beaudart et al., 2016). Nevertheless, these tools are not used in community-based settings because of the high equipment costs and the requirement for highly trained personnel to use the equipment. In addition, the weakness of BIA is its portability and location restrictions. Calf circumference measurement is a noninvasive and straightforward assessment method that is easy to use in community settings. Besides our results, calf circumference was significant positively correlated DXA-based ASM (r = 0.81 in male) (Kawakami et al., 2014). In this study, the results of CT-based SMI and calf circumference (approximately 0.48, *p* < 0.01), and CT-based SMI and BIA-based SMI (approximately 0.38, *p* < 0.01) were correlated (Figure 4). CT SMI was better associated with calf circumference than BIA-based SMI. Our results showed that calf circumference may be used to measure muscle mass in sarcopenia.

**FIGURE 5:**
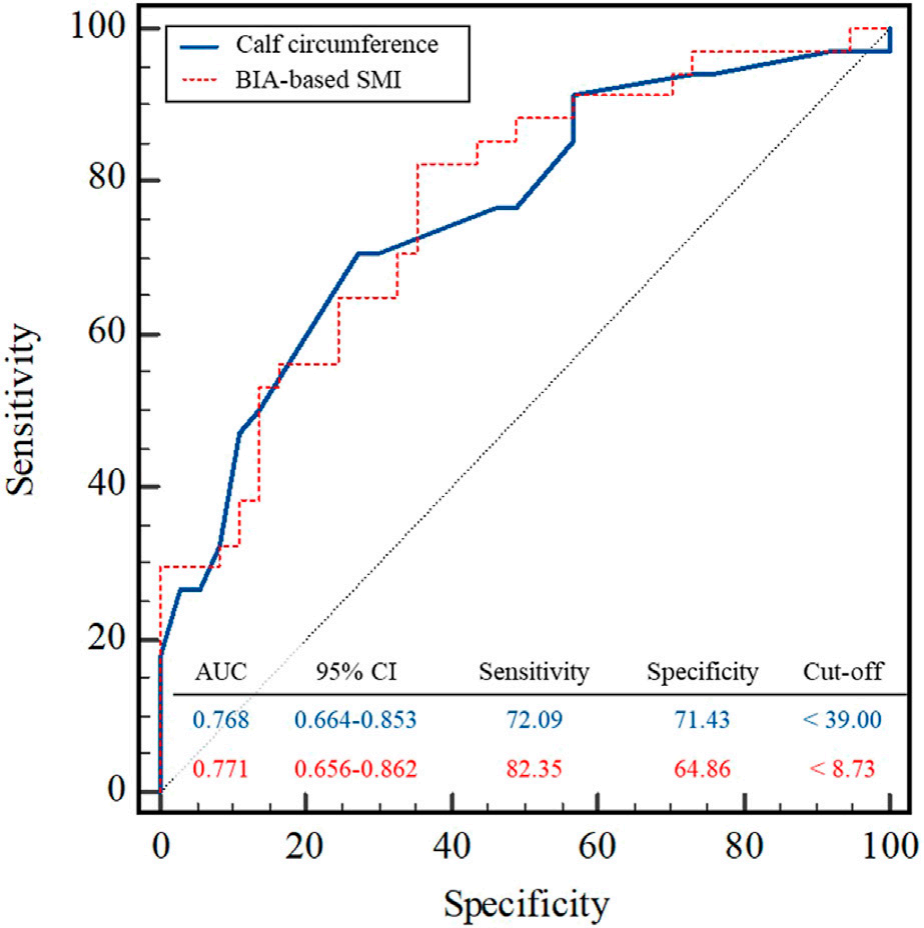
ROC curves of the calf circumference and BIA-based SMI in the sarcopenia group in relation to three variables. BIA, bioelectrical impedance analysis; SMI, skeletal muscle mass index; CI; confidence interval.

The cut-off for calf circumference was defined as <34 cm, and that for SMI was <7 kg/m^2^ in men according to the AWGS and EWGSOP (Cruz-Jentoft et al., 2019; Chen et al., 2020). However, the present results identified cut-offs for calf circumference of <39 cm and SMI of <8.73 kg/m^2^ in our study (Figure 5). This was because the present study examined a relatively young and healthy adult population. Calf circumference and BIA-based SMI showed similar results with respect to the AUC in the ROC analysis. However, BIA-based SMI was more sensitive to CT-based SMI than calf circumference. However, calf circumference had a specificity (71.43 and 64.86) better than that of BIA-based SMI while having a similar sensitivity (72.09 and 82.35). Finally, calf circumference should considered as a tool for assessing muscle mass in the diagnosis of sarcopenia. Calf circumference significantly relates to skeletal muscle mass, and studies recommended to use to diagnose sarcopenia with calf circumference in low-income countries support our argument (González-Correa et al., 2020).

In conclusion, CT-based muscle mass measurement reduces the complexity of the procedure for defining sarcopenia. However, our results were not related to physical function or computed tomography-based sarcopenia. The correlation between CT- and BIA-based SMI was relatively lower in our study, and calf circumference correlated sufficiently with CT-based SMI and grip strength. Therefore, calf circumference appears to be a potential substitute marker for muscle mass index measured using CT and might be a simple screening tool for sarcopenia. It is expected to replace the complex and/or expensive SMI measurement methods.

Our study had some limitations. First, in the early research design, testosterone and sarcopenia were thought to relate. Therefore, this study only included men. However, the relatively young males showed no difference in testosterone. In the future, if there were female participants, the results may have been different. Second, the sample size was small. Follow-up studies should be conducted with a large sample size and include female participants.

## Data Availability

The raw data supporting the conclusions of this article will be made available by the authors, without undue reservation.

## References

[B1] Baumgartner R. N. Koehler K. M. Gallagher D. Romero L. Heymsfield S. B. Ross R. R. (1998). Epidemiology of sarcopenia among the elderly in new Mexico. Am. J. Epidemiol. 147, 755–763. Pmid: 9554417. 10.1093/oxfordjournals.aje.a009520 9554417

[B2] Baumgartner R. N. Waters D. L. Gallagher D. Morley J. E. Garry P. J. (1999). Predictors of skeletal muscle mass in elderly men and women. Mech. Ageing Dev. 107, 123–136. 10.1016/S0047-6374(98)00130-4 10220041

[B3] Beaudart C. Mccloskey E. Bruyêre O. Cesari M. Rolland Y. Rizzoli R. (2016). Sarcopenia in daily practice: Assessment and management. BMC Geriatr. 16, 170. 10.1186/S12877-016-0349-4 27716195 PMC5052976

[B4] Chen L.-K. Liu L.-K. Woo J. Assantachai P. Auyeung T.-W. Bahyah K. S. (2014). Sarcopenia in Asia: Consensus report of the asian working group for sarcopenia. J. Am. Med. Dir. Assoc. 15, 95–101. 10.1016/J.Jamda.2013.11.025 24461239

[B5] Chen L.-K. Woo J. Assantachai P. Auyeung T.-W. Chou M.-Y. Iijima K. (2020). Asian working group for sarcopenia: 2019 consensus update on sarcopenia diagnosis and treatment. J. Am. Med. Dir. Assoc. 21, 300–307. 10.1016/J.Jamda.2019.12.012 32033882

[B6] Cruz-Jentoft A. J. Baeyens J. P. Bauer J. M. Boirie Y. Cederholm T. Landi F. (2010). Sarcopenia: European consensus on definition and diagnosis: Report of the European working group on sarcopenia in older People. Age Ageing 39, 412–423. 10.1093/Ageing/Afq034 20392703 PMC2886201

[B7] Cruz-Jentoft A. J. Bahat G. Bauer J. Boirie Y. Bruyêre O. Cederholm T. (2019). Sarcopenia: Revised European consensus on definition and diagnosis. Age Ageing 48, 16–31. 10.1093/Ageing/Afy169 30312372 PMC6322506

[B8] Du Y. No J. K. (2017). Sarcopenia: Nutrition and related diseases. culinarysciencehospitalityresearch. 23, 66–78. 10.20878/Cshr.2017.23.1.008

[B9] Du Y. Oh C. No J.-K. (2017). Osteosarcopenic obesity in elderly: The cascade of bone, muscle, and fat in inflammatory process. Culin. Sci. Hosp. Res. 23, 173–183. 10.20878/Cshr.2017.23.6.019

[B10] Du Y. Oh C. No J. (2018). Associations between sarcopenia and metabolic risk factors: A systematic review and meta-analysis. J. Obes. Metab. Syndr. 27, 175–185. 10.7570/Jomes.2018.27.3.175 31089560 PMC6504194

[B11] Evans W. J. Chumlea W. C. Guo S. S. Vellas B. Guigoz Y. (1995). Techniques of assessing muscle mass and function (sarcopenia) for epidemiological studies of the elderly. J. Gerontol. A Biol. Sci. Med. Sci. 50, 45–51. 10.1093/Gerona/50a.Special_Issue.45 7493217

[B12] Fearon K. Strasser F. Anker S. D. Bosaeus I. Bruera E. Fainsinger R. L. (2011). Definition and classification of cancer cachexia: An international consensus. Lancet. Oncol. 12, 489–495. 10.1016/S1470-2045(10)70218-7 21296615

[B13] González-Correa C. Pineda-Zuluaga M. Marulanda-Mejía F. (2020). Skeletal muscle mass by bioelectrical impedance analysis and calf circumference for sarcopenia diagnosis. J. Electr. Bioimpedance 11 (1), 57–61. 10.2478/Joeb-2020-0009 33584904 PMC7531101

[B14] Gregor M. F. Hotamisligil G. S. (2011). Inflammatory mechanisms in obesity. Annu. Rev. Immunol. 29, 415–445. 10.1146/Annurev-Immunol-031210-101322 21219177

[B15] Janssen I. Heymsfield S. B. Ross R. (2002). Low relative skeletal muscle mass (sarcopenia) in older persons is associated with functional impairment and physical disability. J. Am. Geriatr. Soc. 50, 889–896. 10.1046/J.1532-5415.2002.50216.X 12028177

[B16] Kalinkovich A. Livshits G. (2017). Sarcopenic obesity or obese sarcopenia: A cross talk between age-associated adipose tissue and skeletal muscle inflammation as A main mechanism of the pathogenesis. Ageing Res. Rev. 35, 200–221. 10.1016/J.Arr.2016.09.008 27702700

[B17] Kawakami R. Murakami H. Sanada K. Tanaka N. Sawada S. S. Tabata I. (2014). Calf circumference as A surrogate marker of muscle mass for diagnosing sarcopenia in Japanese men and women. Geriatr. Gerontol. Int. 15, 969–976. 10.1111/Ggi.12377 25243821

[B18] Montano‐Loza A. J. Angulo P. Meza‐Junco J. Prado C. M. Sawyer M. B. Beaumont C. (2016). Sarcopenic obesity and myosteatosis are associated with higher mortality in patients with cirrhosis. J. Cachexia Sarcopenia Muscle 7, 126–135. 10.1002/Jcsm.12039 27493866 PMC4864157

[B19] Newman A. B. Kupelian V. Visser M. Simonsick E. Goodpaster B. Nevitt M. (2003). Sarcopenia: Alternative definitions and associations with lower extremity function. J. Am. Geriatr. Soc. 51, 1602–1609. 10.1046/J.1532-5415.2003.51534.X 14687390

[B20] Oh J.-Y. Yang Y.-J. Kim B.-S. Kang J.-H. (2007). Validity and reliability of Korean version of international physical activity questionnaire (ipaq) short form. J. Of Korean Acad. Of Fam. Med. 28, 532–541.

[B21] Rosenberg I. H. (1997). Sarcopenia: Origins and clinical relevance. J. Nutr. 127, 990s–991s. 10.1093/Jn/127.5.990s 9164280

[B22] Sattar N. Tan C. Han T. Forster L. Lean M. Shepherd J. (1998). Associations of indices of adiposity with atherogenic lipoprotein subfractions. Int. J. Obes. Relat. Metab. Disord. 22, 432–439. 10.1038/Sj.Ijo.0800604 9622340

[B23] Shen W. Punyanitya M. Wang Z. Gallagher D. St.-Onge M.-P. Albu J. (2004). Total body skeletal muscle and adipose tissue volumes: Estimation from A single abdominal cross-sectional image. J. Appl. Physiol. 97, 2333–2338. 10.1152/Japplphysiol.00744.2004 15310748

[B24] Stenholm S. Harris T. B. Rantanen T. Visser M. Kritchevsky S. B. Ferrucci L. (2008). Sarcopenic obesity: Definition, cause and consequences. Curr. Opin. Clin. Nutr. Metab. Care 11, 693–700. 10.1097/Mco.0b013e328312c37d 18827572 PMC2633408

[B25] Tang T. Zhuo Y. Xie L. Wang H. Yang M. (2020). Sarcopenia index based on serum creatinine and cystatin C is associated with 3-year mortality in hospitalized older patients. Sci. Rep. 10, 1260–1269. 10.1038/S41598-020-58304-Z 31988356 PMC6985114

[B26] Tyrovolas S. Koyanagi A. Olaya B. Ayuso‐Mateos J. L. Miret M. Chatterji S. (2016). Factors associated with skeletal muscle mass, sarcopenia, and sarcopenic obesity in older adults: A multi‐continent study. J. Cachexia Sarcopenia Muscle 7, 312–321. 10.1002/Jcsm.12076 27239412 PMC4864288

[B27] Walrand S. Guillet C. Salles J. Cano N. Boirie Y. (2011). Physiopathological mechanism of sarcopenia. Clin. Geriatr. Med. 27, 365–385. 10.1016/J.Cger.2011.03.005 21824553

[B28] Wang C. Swerdloff R. S. Iranmanesh A. Dobs A. Snyder P. J. Cunningham G. (2000). The testosterone gel study group, NTransdermal testosterone gel improves sexual function, mood, muscle strength, and body composition parameters in hypogonadal men. J. Clin. Endocrinol. Metab. 85, 2839–2853. 10.1210/Jcem.85.8.6747 10946892

[B29] Zamboni M. Mazzali G. Fantin F. Rossi A. Di Francesco V. (2008). Sarcopenic obesity: A new category of obesity in the elderly. Nutr. Metab. Cardiovasc. Dis. 18, 388–395. 10.1016/J.Numecd.2007.10.002 18395429

[B30] Zamboni M. Turcato E. Santana H. Maggi S. Harris T. B. Pietrobelli A. (1999). The relationship between body composition and physical performance in older women. J. Am. Geriatr. Soc. 47, 1403–1408. 10.1111/j.1532-5415.1999.tb01557.x 10591232

